# Association of lopinavir concentrations with plasma lipid or glucose concentrations in HIV-infected South Africans: a cross sectional study

**DOI:** 10.1186/1742-6405-9-32

**Published:** 2012-10-26

**Authors:** Phumla Z Sinxadi, Helen M McIlleron, Joel A Dave, Peter J Smith, Naomi S Levitt, Gary Maartens

**Affiliations:** 1 Department of Medicine, Division of Clinical Pharmacology, University of Cape Town, Cape Town, South Africa; 2 Department of Medicine, Division of Endocrinology and Diabetes, University of Cape Town, Cape Town, South Africa

**Keywords:** Lopinavir, Hypercholesterolaemia, Hypertriglyceridaemia, Impaired glucose metabolism, Antiretroviral therapy, Pharmacokinetics

## Abstract

**Background:**

Dyslipidaemia and dysglycaemia have been associated with exposure to ritonavir-boosted protease inhibitors. Lopinavir/ritonavir, the most commonly used protease inhibitor in resource-limited settings, often causes dyslipidaemia. There are contradictory data regarding the association between lopinavir concentrations and changes in lipids.

**Aim:**

To investigate associations between plasma lopinavir concentrations and lipid and glucose concentrations in HIV-infected South African adults.

**Methods:**

Participants stable on lopinavir-based antiretroviral therapy were enrolled into a cross-sectional study. After an overnight fast, total cholesterol, triglycerides, and lopinavir concentrations were measured and an oral glucose tolerance test was performed. Regression analyses were used to determine associations between plasma lopinavir concentrations and fasting and 2 hour plasma glucose, fasting cholesterol, and triglycerides concentrations.

**Results:**

There were 84 participants (72 women) with a median age of 36 years. The median blood pressure, body mass index and waist: hip ratio were 108/72 mmHg, 26 kg/m^2^ and 0.89 respectively. The median CD4 count was 478 cells/mm^3^. Median duration on lopinavir was 18.5 months. The median (interquartile range) lopinavir concentration was 8.0 (5.2 to 12.8) μg/mL. Regression analyses showed no significant association between lopinavir pre-dose concentrations and fasting cholesterol (β-coefficient −0.04 (95% CI −0.07 to 0.00)), triglycerides (β-coefficient −0.01 (95% CI −0.04 to 0.02)), fasting glucose (β-coefficient −0.01 (95% CI −0.04 to 0.02)), or 2-hour glucose concentrations (β-coefficient −0.02 (95% CI −0.09 to 0.06)). Lopinavir concentrations above the median were not associated with presence of dyslipidaemia or dysglycaemia.

**Conclusions:**

There was no association between lopinavir plasma concentrations and plasma lipid and glucose concentrations.

## Introduction

Ritonavir-boosted lopinavir (LPV/r) is a potent protease inhibitor (PI) widely used in a second-line antiretroviral regimens in low and middle-income countries [1]. Treatment with LPV/r has been associated with the development of dyslipidaemia [2,3], which is a known risk factor for cardiovascular complications. The Women’s Interagency HIV study showed that dyslipidaemia was more severe in HIV infected women on ritonavir-boosted PI containing antiretroviral therapy (ART), compared with untreated HIV infected women or non-PI based ART [4]. There are conflicting results regarding an association between LPV/r use and the development of insulin resistance and new onset diabetes [5-9]. Furthermore, there are contradictory data as to whether the metabolic toxicity is associated with higher plasma concentrations of either lopinavir or ritonavir [10-12]. Widespread long-term PI use may be limited by these metabolic complications. Data regarding an association between plasma lopinavir and plasma glucose concentration are lacking.

The aim of our study was to investigate whether there is an association between plasma lopinavir and plasma lipids and glucose concentrations. We hypothesized that higher plasma lopinavir concentrations would be associated with higher prevalence of dyslipidaemia and dysglycaemia.

## Materials and methods

### Study design and participants

We conducted a prospective cross sectional study between February 2007 and January 2008. Consecutive ambulatory HIV infected African adults who presented for a routine follow up visit at primary and tertiary level clinics in Cape Town were recruited. Participants were eligible if they were on lopinavir-based therapy for a minimum of six months. Participants with renal or hepatic disease, active opportunistic infection, diabetes, history of diabetes or dyslipidaemia and self-reported non-adherence were excluded. The study was approved by the University of Cape Town research ethics committee.

### Clinical and laboratory evaluations

After obtaining informed consent, we instructed the participants to undergo an overnight fast and to note the time of taking the evening dose of lopinavir on the day proceeding the study day. On the study day, participants underwent an oral glucose tolerance tests (OGTT). Blood was drawn at 0 and 120 minutes after ingestion of 75g glucose in 250ml water, into heparinised or sodium fluoride tubes as appropriate, and kept on ice until centrifugation within 4 hours. Plasma lopinavir concentrations and serum fasting glucose, cholesterol, and triglyceride concentrations were determined from the 0 minute samples of the OGTT. The morning doses of ART were given after 2 hour glucose samples were taken.

Lopinavir drug concentrations were analyzed by fully validated methods using liquid chromatography-tandem mass spectrometry (LC-MS/MS) on an Applied Biosystems MDS Sciex API 4000 tandem mass spectrometer at our ISO17025 compliant and accredited analytical laboratory as previously described [13]. The assay range was 0.05 to 20 μg/mL. Accuracy ranged from 94 to 103%. Any samples with lopinavir concentrations below the limit of quantification (0.05 μg/mL) were fixed to 0.025 μg/mL. Serum glucose and lipid concentrations were determined by standard methods using the ACE Alera Clinical Chemistry System (Alfa Wassermann Diagnostic Technologies, Woerden, Netherlands). Impaired fasting glucose (IFG) and impaired glucose tolerance (IGT) were defined according to the American Diabetes Association criteria [14]. Hypercholesterolaemia and hypertriglyceridaemia were defined according to the NCEP III criteria [15].

We reviewed medical records to determine duration on antiretroviral therapy, viral load and CD4 counts. Self reported adherence was determined using a validated 4-day adherence questionnaire administered by trained field workers [16]. Basic anthropometric measurements were measured by a biokineticist.

### Pharmacokinetic and statistical analysis

The plasma lopinavir concentrations were obtained from 0 minute of the OGTT. For data points below the limit of quantification (0.05 μg/mL), the lopinavir concentrations were fixed to 0.025 μg/mL. The associations between lopinavir concentrations and glucose, triglycerides, and cholesterol were determined using univariate linear regression and multivariate analyses. Logistic regression was used to determine associations between lopinavir concentrations above the median and metabolic complications (hypercholesterolaemia, hypertriglyceridaemia and dysglycaemia).

### Sample size calculation

A previous study found a positive correlation (rho) of 0.32 between lopinavir trough levels and triglyceride levels [10]. When the sample size is 50, the linear regression test of rho=0 (alpha= 0.050 two-sided) for normally distributed triglyceride concentrations, will have 80% power to detect a rho of 0.375.

## Results

**Figure 1:**
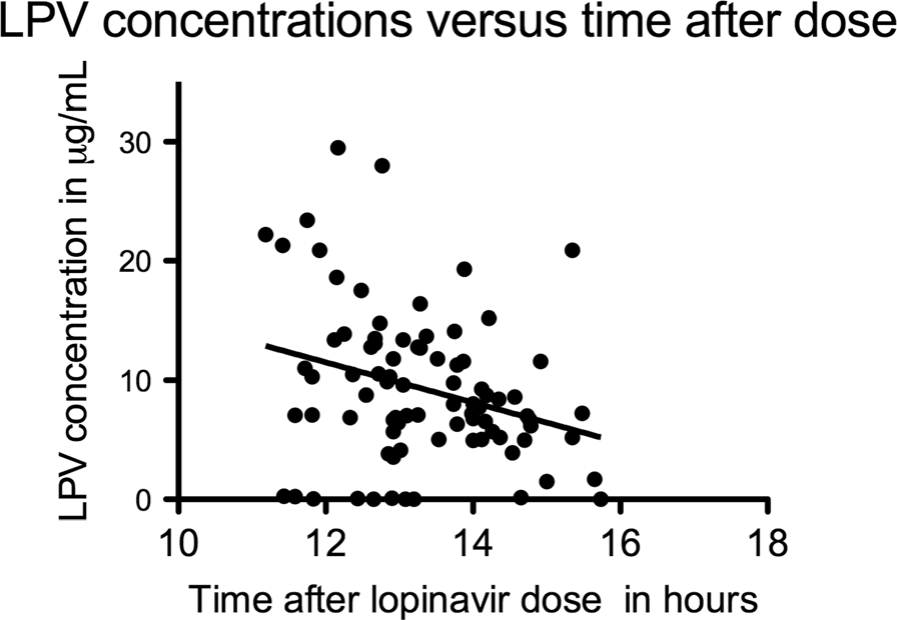
**Scatter plot of lopinavir concentrations (μg/mL) and the time after the last lopinavir dose (hours).** The black dots denote individual lopinavir concentrations plotted against the time after the last unobserved lopinavir dose (dose to sampling time) from 84 participants. The regression line shows negative correlation between the lopinavir concentrations and the dose to sampling time (Spearman rho (95%CI)= −0.28(−0.47 to −0.06), p-value 0.01).

**Figure 2:**
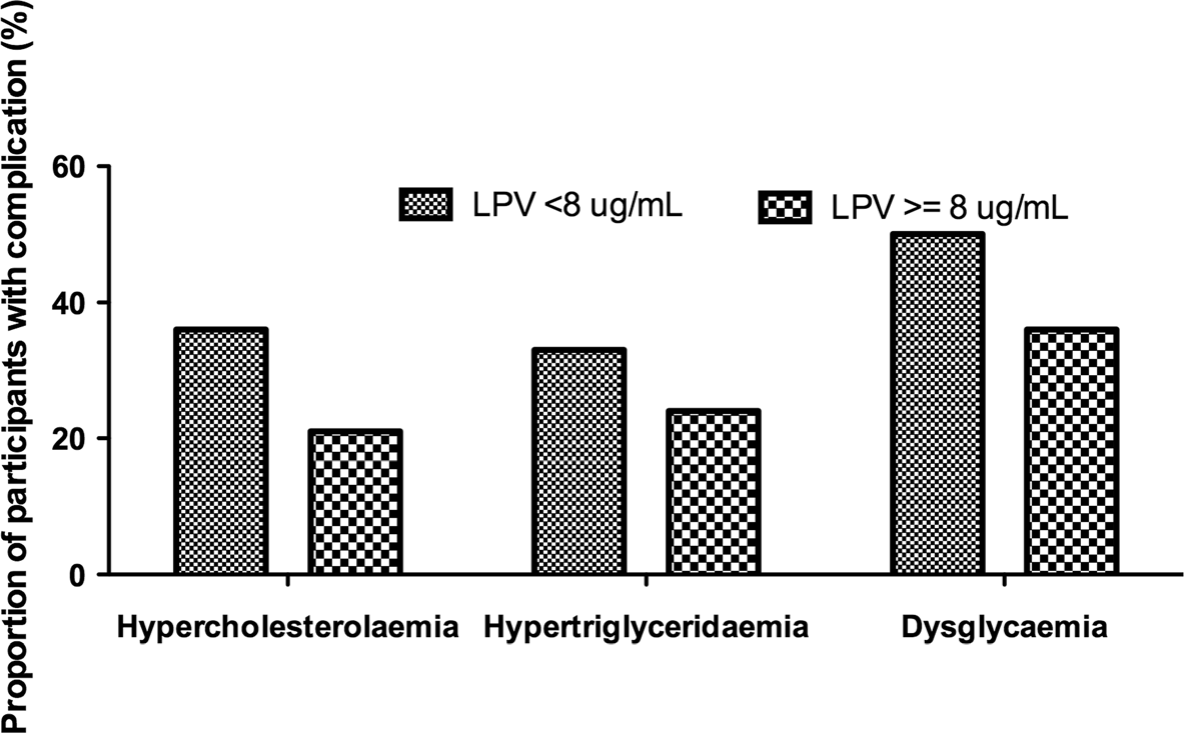
**Proportion of participants with dyslipidaemia and dysglycaemia categorized by the lopinavir concentration below or above the median (8μg/mL).** The bar graphs show the proportion of participants (%) who had hypercholesterolaemia, hypertriglyceridaemia and dysglycaemia in 42 participants with lopinavir below the median (fine checked bars) and in 42 participants with lopinavir concentrations equal to, or above the median (course checked bars). There was no association between the lopinavir concentrations above the median and the metabolic complications. The odds ratios for lopinavir above the median and the complications were: hypercholesterolaemia OR (95%CI): 0.49 (0.19 to 1.30); hypertriglyceridaemia OR (95%CI): 0.56 (0.29 to 1.33); dysglycaemia OR (95 CI): 0.63 (0.24 to 1.63).

Of 93 participants enrolled for the study, nine were excluded due to missing anthropometric and metabolic data or inaccurate dose-sampling time. The baseline characteristics of the 84 participants included in the analysis are in Table 1. The majority were women, reflecting the epidemic seen in our clinical practice. All participants reported 100% adherence. The prevalence of hypercholesterolaemia, hypertriglyceridaemia, impaired fasting glucose, impaired glucose tolerance, and diabetes were 29%, 29%, 25%, 14% and 4% respectively. The plasma LPV concentrations are shown in Figure 1. The median (IQR) time after dose was 13.2 (12.6 to 14.1) hours. There were ten participants whose lopinavir concentrations were below 1 μg/mL (12%). Of these, 4 were virologically suppressed, 4 had virologic failure, and 2 had missing viral load data. As there was a significant association between time after dose and lopinavir concentrations, the time after dose was included in the multivariate regression analyses investigating the association between lopinavir concentrations and metabolic parameters. There were no significant associations between lopinavir concentrations and lipid and glucose concentrations on simple regression analyses, or after adjusting for age, sex, time after dose, and duration on lopinavir (Table 2). The lack of association between lopinavir concentrations and the metabolic parameters persisted when the 10 participants with lopinavir concentrations below 1 μg/mL (as they were probably non-adherent) were excluded (data not shown). Sixty one percent of participants were on zidovudine and didanosine combination (Table 1), as recommended by the South African antiretroviral guidelines at the time. However, the different NRTI backbone was not associated with any of the outcomes evaluated. For example, for triglycerides, in the participants of the same age, sex, duration on lopinavir, and with same lopinavir concentration taken at the same time after dose, there was no significant difference between those who were on zidovudine/didanosine combination compared to other NRTI combinations [mean difference −0.22(−0.65 to 0.21) mmol/L, p value =0.31]. Similarly, there was no association with NRTI backbone and cholesterol if there all parameters remained the same [mean difference −0.18(−0.73 to 0.37) mmol/L, p value =0.51]. There was no association between lopinavir concentrations above the median (8 μg/mL) and dyslipidaemia or dysglycaemia (see Figure 2).

**Table 1:** Study population characteristics, N=84

**Variable**	**Median (IQR) or n/N(%)**
Age (years)	36 (32–42)
Female n/N (%)	72/84 (86)
Race n/N (%)	
Black	84/84 (100)
Weight (kg)	69 (60–82)
Body mass index (kg/m^2^)	26 (23–32)
Waist: hip ratio	0.88 (0.82-0.94)
Skin fold thickness (mm)	
Triceps	17 (11–25)
Abdomen	24 (17–40)
Thigh	30 (17–46)
Calf	16 (9–21)
Blood pressure mmHg	108/72 (102/66-119/77)
CD4 count (cells/ mm^3^)	
Pre-ART	103 (37–140)
Current	468 (291–623)
Current viral load	
Proportion with <400 copies/mL (%)*	64/74 (86)
Duration on lopinavir (months)	19 (9–29)
Concurrent ART n/N (%)	
Zidovudine/didanosine	51/84 (61)
Zidovudine/lamivudine	17/84 (20)
Stavudine/lamivudine	10/84 (12)
Efavirenz	4/84 (5)
Nevirapine	2/84 (2)
Metabolic parameters (mmol/L)	
Fasting cholesterol	4.3 (3.7 to 5.3)
Fasting triglycerides	1.3 (0.9 to 1.8)
Fasting glucose	5.2 (4.7 to 5.7)
2 hour glucose	6.3 (5.4 to 8.1)

* 10 participants had missing current viral load data.

**Table 2:** Association between lopinavir trough concentrations and lipid and glucose concentrations

	**Univariate analyses**		**Multivariate analyses**	
**Variable**	**Beta coefficient (95%CI)**	**P value**	**Beta coefficient (95% CI)**	**P value**
Total cholesterol	−0.04 (−0.07 to 0.00)	0.07	−0.02 (−0.06 to 0.01)	0.21
Triglycerides	−0.01 (−0.04 to 0.02)	0.53	−0.00 (−0.03 to 0.02)	0.86
Fasting glucose	−0.01 (−0.04 to 0.02)	0.44	−0.00 (−0.03 to 0.02)	0.78
2 hour glucose	−0.02 (−0.09 to 0.06)	0.64	0.00 (−0.05 to 0.06)	0.85

Adjusted for time after dose, age, sex and duration on lopinavir. In the multivariate model for 2 hour glucose, time after dose was an independent predictor [β-coefficient 0.56 (95% CI 0.23 to 0.88), p= 0.001].

## Discussion

To our knowledge, this is the first study to investigate the association between lopinavir concentrations and serum glucose concentrations, and the first to investigate associations between lopinavir concentrations and serum lipids in a South African population. Despite a high prevalence of dyslipidaemia (29%) and dysglycaemia (42%) in 84 black South African HIV-infected adults treated with ritonavir-boosted lopinavir for a median duration of 19 months, we found no association between plasma lopinavir concentrations and lipid or glucose concentrations. There was also no significant association between the lopinavir concentrations above the median, and hypercholesterolaemia, hypertriglyceridaemia or dysglycaemia.

We found the median lopinavir concentration was 8 μg/mL, which is higher than reported elsewhere [17,18], but comparable to the trough concentrations after observed doses in a study conducted by our group from the same community [19]. Lopinavir pharmacokinetics demonstrate considerable interindividual variability, which may affect treatment outcomes. At least part of this variability may be explained by host genetic factors. Associations between human genetic variants and lopinavir exposure are incompletely understood and need to be explored.

The lack of an association between lopinavir concentrations and lipid or glucose concentrations that we found can be explained as follows: First, like all protease inhibitors, lopinavir exerts its antiviral activity intracellularly, and the plasma and intracellular half-lives are different [20]. However, the positive correlation between lopinavir plasma and intracellular concentrations reported at 4 weeks was not sustained at 24 weeks of treatment [20]. More importantly, the mechanism of toxicity associated with protease inhibitor use, including lopinavir, is still poorly understood, but is thought to be related to interference with some cellular endogenous processes. For example, lopinavir binds to lipoprotein receptor related protein (LPR), impairing hepatic chylomicron uptake and triglyceride clearance by LPR –lipoprotein lipase complex, causing hyperlipidaemia [21]. In susceptible individuals, the resulting hyperlipidaemia may induce diabetes [21]. Second, clinical manifestations of LPV/r toxicity are also influenced by host susceptibility such as age, sex, weight, race, advanced HIV disease, concomitant ART, higher baseline triglyceride or cholesterol concentrations, and genetic susceptibility [2,22,23]. Finally, we hypothesize that the if the association between plasma lopinavir concentrations and toxicity exists, it exists in much lower concentrations, as the dose–response curve is likely to be flat at the concentrations found in our study. Therefore, the influence of lopinavir plasma concentrations on lipids at therapeutic doses, is likely to be small and larger studies are needed to detect the small differences. Furthermore, recent data suggests that lopinavir does not impair insulin sensitivity [7,8], and therefore, the lack of an association between lopinavir and glucose concentrations is not surprising.

Our findings are in contrast with findings from a small study conducted in 19 patients, which reported that lopinavir trough concentrations were higher in three patients experiencing grade 3 or 4 lipid elevations [11]. A second larger study (n=126) found that patients with fasting triglyceride concentrations above the median had higher lopinavir trough concentrations, but no correlation was found between lopinavir and cholesterol concentrations [10]. Four other studies reported findings similar to ours, with no association between lopinavir and lipid concentrations [12,17,24,25]. The older protease inhibitor indinavir is known to cause diabetes [5]. However, after 12 months of treatment with lopinavir, none of the 73 patients included in another study developed diabetes [6]. Studies in healthy volunteers have shown that insulin sensitivity wasn’t altered by lopinavir in healthy HIV negative men [7,8]. However, a single dose study in healthy volunteers showed that lopinavir could inhibit glucose uptake acutely [9]. Data regarding the association between lopinavir concentrations and glucose metabolism are lacking.

Our study has several limitations. First, it is a cross sectional study and we therefore cannot compare lipid or glucose concentrations prior to lopinavir treatment. Second, patients with known diabetes or dyslipidaemia were excluded from the study, and it is possible that lopinavir may have exacerbated a pre-existing metabolic defect. Third, our sample size is relatively small, however, it is larger than most of the previous studies that have investigated this association [11,17,19]. We aimed to examine 50 participants to detect a correlation of 0.375. Our analyses used simple and multivariate regression analyses with various predictors, therefore we continued to slowly recruit eligible participants until the end of the study. Fourth, we investigated associations with pre-dose lopinavir concentrations and metabolic parameters. The pharmacokinetic parameters area under the curve or average steady state would be a better measure of overall drug exposure. Lastly, the last dose of lopinavir was not observed. To minimize recall bias, participants were requested to record the time of last dose on the appointment card for the day before pharmacokinetic sampling.

In conclusion, we did not find an association between lopinavir concentrations and lipid and glucose concentrations. Larger prospective studies are needed to establish whether an association exists between lopinavir concentrations and increasing lipids or glucose metabolism changes.

## Competing interests

The authors declare that they have no competing interests.

## Authors’ contributions

PZS participated in the study design, acquisition of data, data analysis and interpretation and drafted the manuscript. HMM participated in the study design, data interpretation, and critically revised the manuscript. PJS performed the analysis of the samples and helped to draft the manuscript. JAD participated in the study design, acquisition of data, and critically revised the manuscript. NSL participated in study design and critically revised the manuscript. GM conceived of the study, participated in study design, data interpretation and critically revised the manuscript. All authors read and approved the final manuscript.
